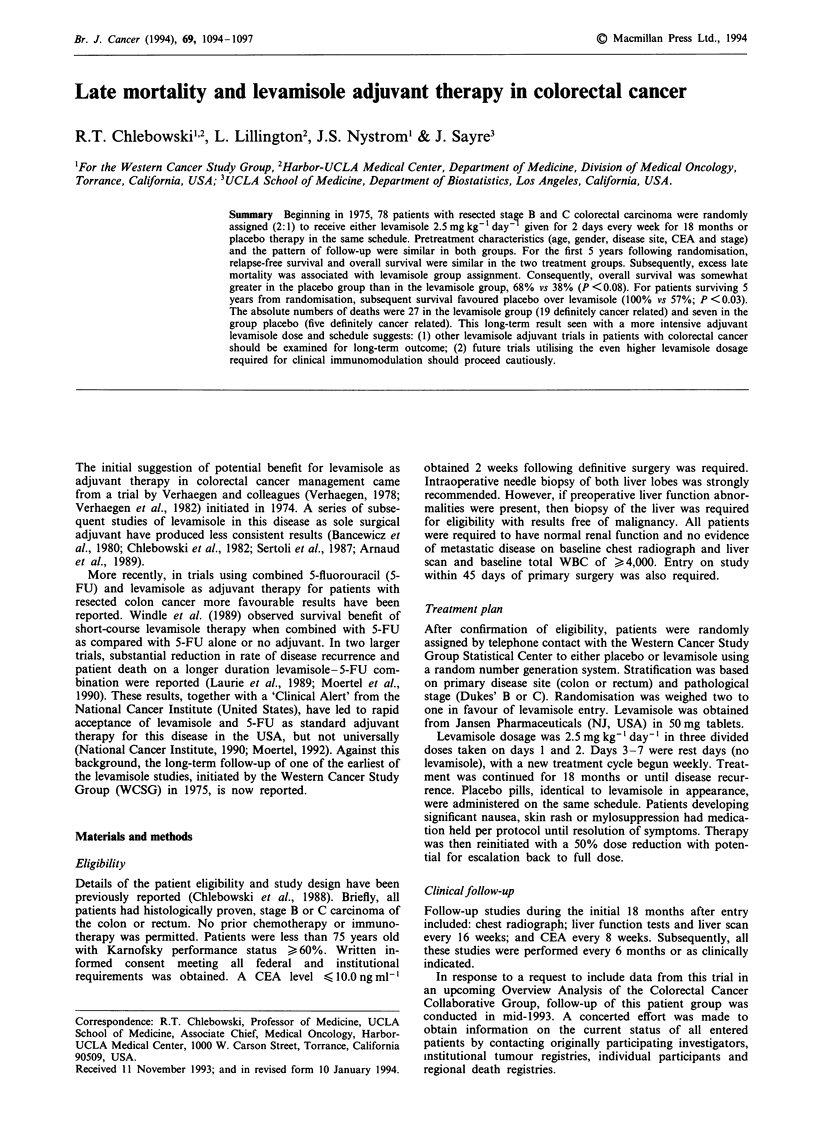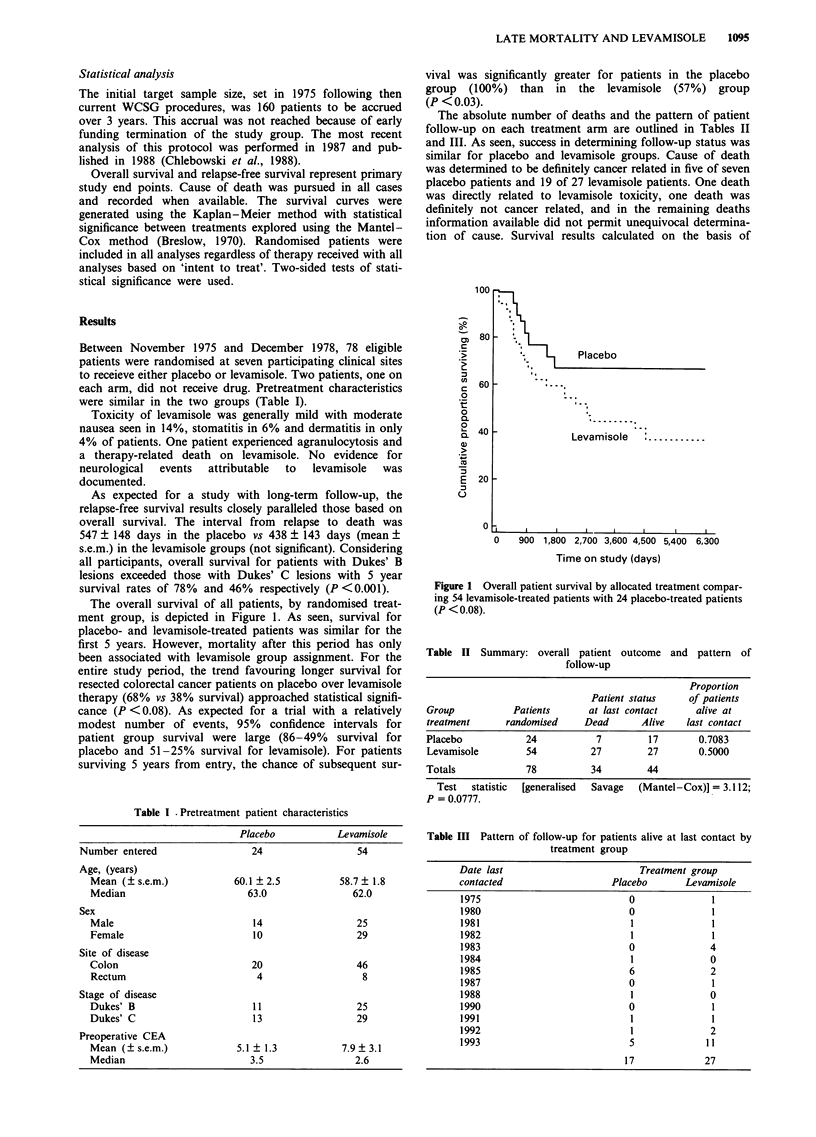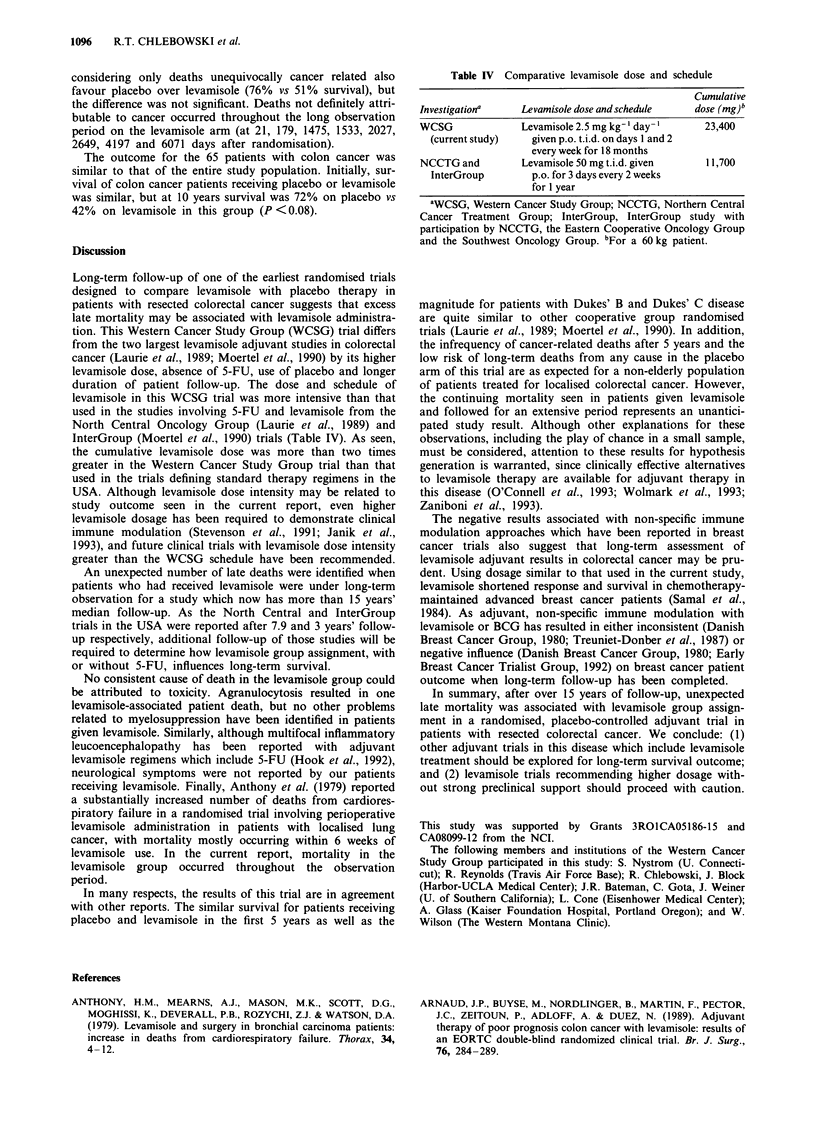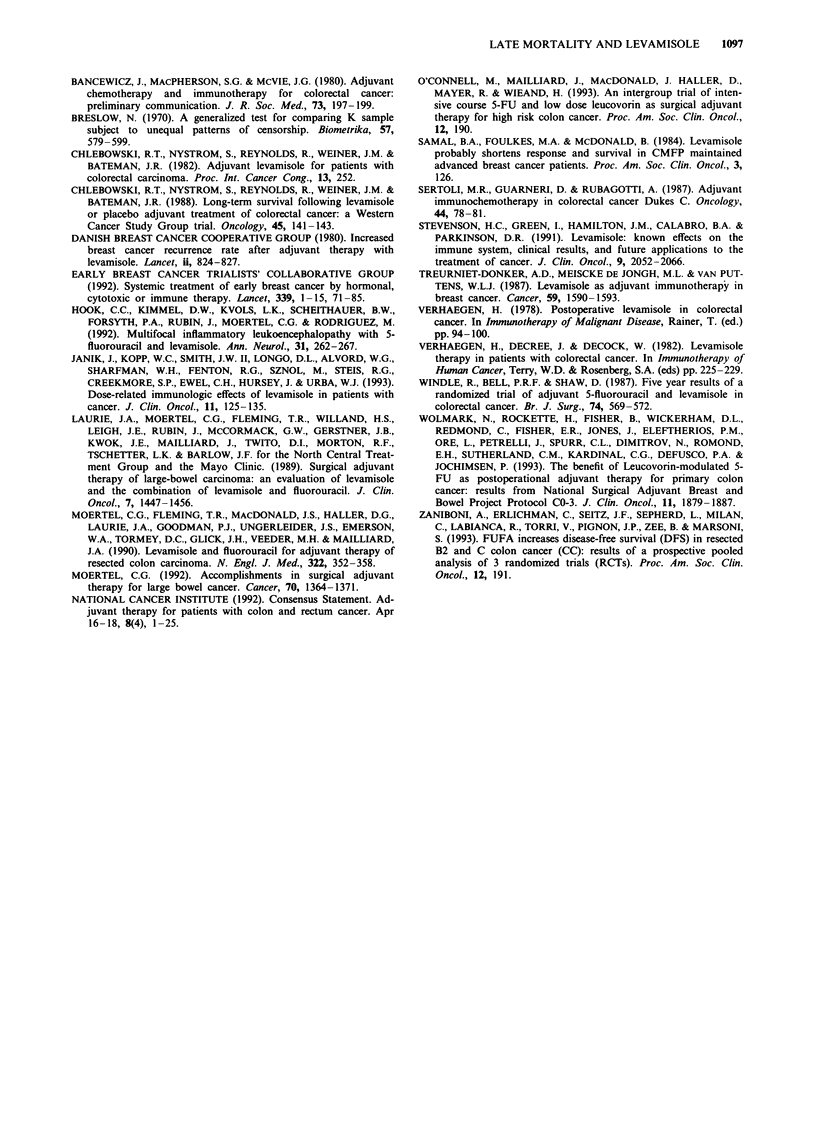# Late mortality and levamisole adjuvant therapy in colorectal cancer.

**DOI:** 10.1038/bjc.1994.214

**Published:** 1994-06

**Authors:** R. T. Chlebowski, L. Lillington, J. S. Nystrom, J. Sayre

**Affiliations:** Harbor-UCLA Medical Center, Department of Medicine, Torrance 90509.

## Abstract

Beginning in 1975, 78 patients with resected stage B and C colorectal carcinoma were randomly assigned (2:1) to receive either levamisole 2.5 mg kg-1 day-1 given for 2 days every week for 18 months or placebo therapy in the same schedule. Pretreatment characteristics (age, gender, disease site, CEA and stage) and the pattern of follow-up were similar in both groups. For the first 5 years following randomisation, relapse-free survival and overall survival were similar in the two treatment groups. Subsequently, excess late mortality was associated with levamisole group assignment. Consequently, overall survival was somewhat greater in the placebo group than in the levamisole group, 68% vs 38% (P < 0.08). For patients surviving 5 years from randomisation, subsequent survival favoured placebo over levamisole (100% vs 57%; P < 0.03). The absolute numbers of deaths were 27 in the levamisole group (19 definitely cancer related) and seven in the group placebo (five definitely cancer related). This long-term result seen with a more intensive adjuvant levamisole dose and schedule suggests: (1) other levamisole adjuvant trials in patients with colorectal cancer should be examined for long-term outcome; (2) future trials utilising the even higher levamisole dosage required for clinical immunomodulation should proceed cautiously.


Br. J. Cancer (1994), 69, 1094–1097 © Macmillan Press Ltd., 1994

### Late mortality and levamisole adjuvant therapy in colorectal cancer

R.T. Chlebowski[1,2], L. Lillington[2], J.S. Nystrom[1] & J. Sayre[3]

[1]*For the Western Cancer Study Group,* [2]*Harbor-UCLA Medical Center, Department of Medicine, Division of Medical Oncology, Torrance, California, USA;* [3]*UCLA School of Medicine, Department of Biostatistics, Los Angeles, California, USA.*

**Summary** Beginning in 1975, 78 patients with resected stage B and C colorectal carcinoma were randomly assigned (2:1) to receive either levamisole 2.5 mg $kg^{-1}$ $day^{-1}$ given for 2 days every week for 18 months or placebo therapy in the same schedule. Pretreatment characteristics (age, gender, disease site, CEA and stage) and the pattern of follow-up were similar in both groups. For the first 5 years following randomisation, relapse-free survival and overall survival were similar in the two treatment groups. Subsequently, excess late mortality was associated with levamisole group assignment. Consequently, overall survival was somewhat greater in the placebo group than in the levamisole group, 68% *vs* 38% ($P < 0.08$). For patients surviving 5 years from randomisation, subsequent survival favoured placebo over levamisole (100% *vs* 57%; $P < 0.03$). The absolute numbers of deaths were 27 in the levamisole group (19 definitely cancer related) and seven in the group placebo (five definitely cancer related). This long-term result seen with a more intensive adjuvant levamisole dose and schedule suggests: (1) other levamisole adjuvant trials in patients with colorectal cancer should be examined for long-term outcome; (2) future trials utilising the even higher levamisole dosage required for clinical immunomodulation should proceed cautiously.

The initial suggestion of potential benefit for levamisole as adjuvant therapy in colorectal cancer management came from a trial by Verhaegen and colleagues (Verhaegen, 1978; Verhaegen *et al.*, 1982) initiated in 1974. A series of subsequent studies of levamisole in this disease as sole surgical adjuvant have produced less consistent results (Bancewicz *et al.*, 1980; Chlebowski *et al.*, 1982; Sertoli *et al.*, 1987; Arnaud *et al.*, 1989).

More recently, in trials using combined 5-fluorouracil (5-FU) and levamisole as adjuvant therapy for patients with resected colon cancer more favourable results have been reported. Windle *et al.* (1989) observed survival benefit of short-course levamisole therapy when combined with 5-FU as compared with 5-FU alone or no adjuvant. In two larger trials, substantial reduction in rate of disease recurrence and patient death on a longer duration levamisole–5-FU combination were reported (Laurie *et al.*, 1989; Moertel *et al.*, 1990). These results, together with a 'Clinical Alert' from the National Cancer Institute (United States), have led to rapid acceptance of levamisole and 5-FU as standard adjuvant therapy for this disease in the USA, but not universally (National Cancer Institute, 1990; Moertel, 1992). Against this background, the long-term follow-up of one of the earliest of the levamisole studies, initiated by the Western Cancer Study Group (WCSG) in 1975, is now reported.

### Materials and methods

### *Eligibility*

Details of the patient eligibility and study design have been previously reported (Chlebowski *et al.*, 1988). Briefly, all patients had histologically proven, stage B or C carcinoma of the colon or rectum. No prior chemotherapy or immunotherapy was permitted. Patients were less than 75 years old with Karnofsky performance status $\geqslant 60\%$. Written informed consent meeting all federal and institutional requirements was obtained. A CEA level $\leqslant 10.0$ ng $ml^{-1}$ obtained 2 weeks following definitive surgery was required. Intraoperative needle biopsy of both liver lobes was strongly recommended. However, if preoperative liver function abnormalities were present, then biopsy of the liver was required for eligibility with results free of malignancy. All patients were required to have normal renal function and no evidence of metastatic disease on baseline chest radiograph and liver scan and baseline total WBC of $\geqslant 4,000$. Entry on study within 45 days of primary surgery was also required.

### *Treatment plan*

After confirmation of eligibility, patients were randomly assigned by telephone contact with the Western Cancer Study Group Statistical Center to either placebo or levamisole using a random number generation system. Stratification was based on primary disease site (colon or rectum) and pathological stage (Dukes' B or C). Randomisation was weighed two to one in favour of levamisole entry. Levamisole was obtained from Jansen Pharmaceuticals (NJ, USA) in 50 mg tablets.

Levamisole dosage was 2.5 mg $kg^{-1}$ $day^{-1}$ in three divided doses taken on days 1 and 2. Days 3–7 were rest days (no levamisole), with a new treatment cycle begun weekly. Treatment was continued for 18 months or until disease recurrence. Placebo pills, identical to levamisole in appearance, were administered on the same schedule. Patients developing significant nausea, skin rash or mylosuppression had medication held per protocol until resolution of symptoms. Therapy was then reinitiated with a 50% dose reduction with potential for escalation back to full dose.

### *Clinical follow-up*

Follow-up studies during the initial 18 months after entry included: chest radiograph; liver function tests and liver scan every 16 weeks; and CEA every 8 weeks. Subsequently, all these studies were performed every 6 months or as clinically indicated.

In response to a request to include data from this trial in an upcoming Overview Analysis of the Colorectal Cancer Collaborative Group, follow-up of this patient group was conducted in mid-1993. A concerted effort was made to obtain information on the current status of all entered patients by contacting originally participating investigators, institutional tumour registries, individual participants and regional death registries.

Correspondence: R.T. Chlebowski, Professor of Medicine, UCLA School of Medicine, Associate Chief, Medical Oncology, Harbor-UCLA Medical Center, 1000 W. Carson Street, Torrance, California 90509, USA.
Received 11 November 1993; and in revised form 10 January 1994.

*Statistical analysis*

The initial target sample size, set in 1975 following then current WCSG procedures, was 160 patients to be accrued over 3 years. This accrual was not reached because of early funding termination of the study group. The most recent analysis of this protocol was performed in 1987 and published in 1988 (Chlebowski *et al.*, 1988).

Overall survival and relapse-free survival represent primary study end points. Cause of death was pursued in all cases and recorded when available. The survival curves were generated using the Kaplan–Meier method with statistical significance between treatments explored using the Mantel–Cox method (Breslow, 1970). Randomised patients were included in all analyses regardless of therapy received with all analyses based on 'intent to treat'. Two-sided tests of statistical significance were used.

### Results

Between November 1975 and December 1978, 78 eligible patients were randomised at seven participating clinical sites to receieve either placebo or levamisole. Two patients, one on each arm, did not receive drug. Pretreatment characteristics were similar in the two groups (Table I).

Toxicity of levamisole was generally mild with moderate nausea seen in 14%, stomatitis in 6% and dermatitis in only 4% of patients. One patient experienced agranulocytosis and a therapy-related death on levamisole. No evidence for neurological events attributable to levamisole was documented.

As expected for a study with long-term follow-up, the relapse-free survival results closely paralleled those based on overall survival. The interval from relapse to death was 547 ± 148 days in the placebo *vs* 438 ± 143 days (mean ± s.e.m.) in the levamisole groups (not significant). Considering all participants, overall survival for patients with Dukes' B lesions exceeded those with Dukes' C lesions with 5 year survival rates of 78% and 46% respectively ($P < 0.001$).

The overall survival of all patients, by randomised treatment group, is depicted in Figure 1. As seen, survival for placebo- and levamisole-treated patients was similar for the first 5 years. However, mortality after this period has only been associated with levamisole group assignment. For the entire study period, the trend favouring longer survival for resected colorectal cancer patients on placebo over levamisole therapy (68% *vs* 38% survival) approached statistical significance ($P < 0.08$). As expected for a trial with a relatively modest number of events, 95% confidence intervals for patient group survival were large (86–49% survival for placebo and 51–25% survival for levamisole). For patients surviving 5 years from entry, the chance of subsequent survival was significantly greater for patients in the placebo group (100%) than in the levamisole (57%) group ($P < 0.03$).

The absolute number of deaths and the pattern of patient follow-up on each treatment arm are outlined in Tables II and III. As seen, success in determining follow-up status was similar for placebo and levamisole groups. Cause of death was determined to be definitely cancer related in five of seven placebo patients and 19 of 27 levamisole patients. One death was directly related to levamisole toxicity, one death was definitely not cancer related, and in the remaining deaths information available did not permit unequivocal determination of cause. Survival results calculated on the basis of

**Table I** Pretreatment patient characteristics

| | *Placebo* | *Levamisole* |
|---|---|---|
| Number entered | 24 | 54 |
| Age, (years) | | |
| Mean (± s.e.m.) | 60.1 ± 2.5 | 58.7 ± 1.8 |
| Median | 63.0 | 62.0 |
| Sex | | |
| Male | 14 | 25 |
| Female | 10 | 29 |
| Site of disease | | |
| Colon | 20 | 46 |
| Rectum | 4 | 8 |
| Stage of disease | | |
| Dukes' B | 11 | 25 |
| Dukes' C | 13 | 29 |
| Preoperative CEA | | |
| Mean (± s.e.m.) | 5.1 ± 1.3 | 7.9 ± 3.1 |
| Median | 3.5 | 2.6 |

**Figure 1** Overall patient survival by allocated treatment comparing 54 levamisole-treated patients with 24 placebo-treated patients ($P < 0.08$).

**Table II** Summary: overall patient outcome and pattern of follow-up

| *Group treatment* | *Patients randomised* | *Patient status at last contact* *Dead* | *Alive* | *Proportion of patients alive at last contact* |
|---|---|---|---|---|
| Placebo | 24 | 7 | 17 | 0.7083 |
| Levamisole | 54 | 27 | 27 | 0.5000 |
| Totals | 78 | 34 | 44 | |

Test statistic [generalised Savage (Mantel–Cox)] = 3.112; $P = 0.0777$.

**Table III** Pattern of follow-up for patients alive at last contact by treatment group

| *Date last contacted* | *Treatment group* *Placebo* | *Levamisole* |
|---|---|---|
| 1975 | 0 | 1 |
| 1980 | 0 | 1 |
| 1981 | 1 | 1 |
| 1982 | 1 | 1 |
| 1983 | 0 | 4 |
| 1984 | 1 | 0 |
| 1985 | 6 | 2 |
| 1987 | 0 | 1 |
| 1988 | 1 | 0 |
| 1990 | 0 | 1 |
| 1991 | 1 | 1 |
| 1992 | 1 | 2 |
| 1993 | 5 | 11 |
| | 17 | 27 |

considering only deaths unequivocally cancer related also favour placebo over levamisole (76% *vs* 51% survival), but the difference was not significant. Deaths not definitely attributable to cancer occurred throughout the long observation period on the levamisole arm (at 21, 179, 1475, 1533, 2027, 2649, 4197 and 6071 days after randomisation).

The outcome for the 65 patients with colon cancer was similar to that of the entire study population. Initially, survival of colon cancer patients receiving placebo or levamisole was similar, but at 10 years survival was 72% on placebo *vs* 42% on levamisole in this group ($P < 0.08$).

### Discussion

Long-term follow-up of one of the earliest randomised trials designed to compare levamisole with placebo therapy in patients with resected colorectal cancer suggests that excess late mortality may be associated with levamisole administration. This Western Cancer Study Group (WCSG) trial differs from the two largest levamisole adjuvant studies in colorectal cancer (Laurie *et al.*, 1989; Moertel *et al.*, 1990) by its higher levamisole dose, absence of 5-FU, use of placebo and longer duration of patient follow-up. The dose and schedule of levamisole in this WCSG trial was more intensive than that used in the studies involving 5-FU and levamisole from the North Central Oncology Group (Laurie *et al.*, 1989) and InterGroup (Moertel *et al.*, 1990) trials (Table IV). As seen, the cumulative levamisole dose was more than two times greater in the Western Cancer Study Group trial than that used in the trials defining standard therapy regimens in the USA. Although levamisole dose intensity may be related to study outcome seen in the current report, even higher levamisole dosage has been required to demonstrate clinical immune modulation (Stevenson *et al.*, 1991; Janik *et al.*, 1993), and future clinical trials with levamisole dose intensity greater than the WCSG schedule have been recommended.

**Table IV** Comparative levamisole dose and schedule

| *Investigation*[a] | *Levamisole dose and schedule* | *Cumulative dose (mg)*[b] |
|---|---|---|
| WCSG (current study) | Levamisole 2.5 mg $kg^{-1}$ $day^{-1}$ given p.o. t.i.d. on days 1 and 2 every week for 18 months | 23,400 |
| NCCTG and InterGroup | Levamisole 50 mg t.i.d. given p.o. for 3 days every 2 weeks for 1 year | 11,700 |

[a]WCSG, Western Cancer Study Group; NCCTG, Northern Central Cancer Treatment Group; InterGroup, InterGroup study with participation by NCCTG, the Eastern Cooperative Oncology Group and the Southwest Oncology Group. [b]For a 60 kg patient.

An unexpected number of late deaths were identified when patients who had received levamisole were under long-term observation for a study which now has more than 15 years' median follow-up. As the North Central and InterGroup trials in the USA were reported after 7.9 and 3 years' follow-up respectively, additional follow-up of those studies will be required to determine how levamisole group assignment, with or without 5-FU, influences long-term survival.

No consistent cause of death in the levamisole group could be attributed to toxicity. Agranulocytosis resulted in one levamisole-associated patient death, but no other problems related to myelosuppression have been identified in patients given levamisole. Similarly, although multifocal inflammatory leucoencephalopathy has been reported with adjuvant levamisole regimens which include 5-FU (Hook *et al.*, 1992), neurological symptoms were not reported by our patients receiving levamisole. Finally, Anthony *et al.* (1979) reported a substantially increased number of deaths from cardiorespiratory failure in a randomised trial involving perioperative levamisole administration in patients with localised lung cancer, with mortality mostly occurring within 6 weeks of levamisole use. In the current report, mortality in the levamisole group occurred throughout the observation period.

In many respects, the results of this trial are in agreement with other reports. The similar survival for patients receiving placebo and levamisole in the first 5 years as well as the magnitude for patients with Dukes' B and Dukes' C disease are quite similar to other cooperative group randomised trials (Laurie *et al.*, 1989; Moertel *et al.*, 1990). In addition, the infrequency of cancer-related deaths after 5 years and the low risk of long-term deaths from any cause in the placebo arm of this trial are as expected for a non-elderly population of patients treated for localised colorectal cancer. However, the continuing mortality seen in patients given levamisole and followed for an extensive period represents an unanticipated study result. Although other explanations for these observations, including the play of chance in a small sample, must be considered, attention to these results for hypothesis generation is warranted, since clinically effective alternatives to levamisole therapy are available for adjuvant therapy in this disease (O'Connell *et al.*, 1993; Wolmark *et al.*, 1993; Zaniboni *et al.*, 1993).

The negative results associated with non-specific immune modulation approaches which have been reported in breast cancer trials also suggest that long-term assessment of levamisole adjuvant results in colorectal cancer may be prudent. Using dosage similar to that used in the current study, levamisole shortened response and survival in chemotherapy-maintained advanced breast cancer patients (Samal *et al.*, 1984). As adjuvant, non-specific immune modulation with levamisole or BCG has resulted in either inconsistent (Danish Breast Cancer Group, 1980; Treuniet-Donber *et al.*, 1987) or negative influence (Danish Breast Cancer Group, 1980; Early Breast Cancer Trialist Group, 1992) on breast cancer patient outcome when long-term follow-up has been completed.

In summary, after over 15 years of follow-up, unexpected late mortality was associated with levamisole group assignment in a randomised, placebo-controlled adjuvant trial in patients with resected colorectal cancer. We conclude: (1) other adjuvant trials in this disease which include levamisole treatment should be explored for long-term survival outcome; and (2) levamisole trials recommending higher dosage without strong preclinical support should proceed with caution.

This study was supported by Grants 3RO1CA05186-15 and CA08099-12 from the NCI.

The following members and institutions of the Western Cancer Study Group participated in this study: S. Nystrom (U. Connecticut); R. Reynolds (Travis Air Force Base); R. Chlebowski, J. Block (Harbor-UCLA Medical Center); J.R. Bateman, C. Gota, J. Weiner (U. of Southern California); L. Cone (Eisenhower Medical Center); A. Glass (Kaiser Foundation Hospital, Portland Oregon); and W. Wilson (The Western Montana Clinic).

### References

ANTHONY, H.M., MEARNS, A.J., MASON, M.K., SCOTT, D.G., MOGHISSI, K., DEVERALL, P.B., ROZYCHI, Z.J. & WATSON, D.A. (1979). Levamisole and surgery in bronchial carcinoma patients: increase in deaths from cardiorespiratory failure. *Thorax*, **34**, 4-12.

ARNAUD, J.P., BUYSE, M., NORDLINGER, B., MARTIN, F., PECTOR, J.C., ZEITOUN, P., ADLOFF, A. & DUEZ, N. (1989). Adjuvant therapy of poor prognosis colon cancer with levamisole: results of an EORTC double-blind randomized clinical trial. *Br. J. Surg.*, **76**, 284-289.

BANCEWICZ, J., MACPHERSON, S.G. & MCVIE, J.G. (1980). Adjuvant chemotherapy and immunotherapy for colorectal cancer: preliminary communication. *J. R. Soc. Med.*, **73**, 197–199.

BRESLOW, N. (1970). A generalized test for comparing K sample subject to unequal patterns of censorship. *Biometrika*, **57**, 579–599.

CHLEBOWSKI, R.T., NYSTROM, S., REYNOLDS, R., WEINER, J.M. & BATEMAN, J.R. (1982). Adjuvant levamisole for patients with colorectal carcinoma. *Proc. Int. Cancer Cong.*, **13**, 252.

CHLEBOWSKI, R.T., NYSTROM, S., REYNOLDS, R., WEINER, J.M. & BATEMAN, J.R. (1988). Long-term survival following levamisole or placebo adjuvant treatment of colorectal cancer: a Western Cancer Study Group trial. *Oncology*, **45**, 141–143.

DANISH BREAST CANCER COOPERATIVE GROUP (1980). Increased breast cancer recurrence rate after adjuvant therapy with levamisole. *Lancet*, **ii**, 824–827.

EARLY BREAST CANCER TRIALISTS' COLLABORATIVE GROUP (1992). Systemic treatment of early breast cancer by hormonal, cytotoxic or immune therapy. *Lancet*, **339**, 1–15, 71–85.

HOOK, C.C., KIMMEL, D.W., KVOLS, L.K., SCHEITHAUER, B.W., FORSYTH, P.A., RUBIN, J., MOERTEL, C.G. & RODRIGUEZ, M. (1992). Multifocal inflammatory leukoencephalopathy with 5-fluorouracil and levamisole. *Ann. Neurol.*, **31**, 262-267.

JANIK, J., KOPP, W.C., SMITH, J.W. II, LONGO, D.L., ALVORD, W.G., SHARFMAN, W.H., FENTON, R.G., SZNOL, M., STEIS, R.G., CREEKMORE, S.P., EWEL, C.H., HURSEY, J. & URBA, W.J. (1993). Dose-related immunologic effects of levamisole in patients with cancer. *J. Clin. Oncol.*, **11**, 125–135.

LAURIE, J.A., MOERTEL, C.G., FLEMING, T.R., WILLAND, H.S., LEIGH, J.E., RUBIN, J., MCCORMACK, G.W., GERSTNER, J.B., KWOK, J.E., MAILLIARD, J., TWITO, D.I., MORTON, R.F., TSCHETTER, L.K. & BARLOW, J.F. for the North Central Treatment Group and the Mayo Clinic. (1989). Surgical adjuvant therapy of large-bowel carcinoma: an evaluation of levamisole and the combination of levamisole and fluorouracil. *J. Clin. Oncol.*, **7**, 1447–1456.

MOERTEL, C.G., FLEMING, T.R., MACDONALD, J.S., HALLER, D.G., LAURIE, J.A., GOODMAN, P.J., UNGERLEIDER, J.S., EMERSON, W.A., TORMEY, D.C., GLICK, J.H., VEEDER, M.H. & MAILLIARD, J.A. (1990). Levamisole and fluorouracil for adjuvant therapy of resected colon carcinoma. *N. Engl. J. Med.*, **322**, 352–358.

MOERTEL, C.G. (1992). Accomplishments in surgical adjuvant therapy for large bowel cancer. *Cancer*, **70**, 1364–1371.

NATIONAL CANCER INSTITUTE (1992). Consensus Statement. Adjuvant therapy for patients with colon and rectum cancer. Apr 16–18, **8**(4), 1–25.

O'CONNELL, M., MAILLIARD, J., MACDONALD, J. HALLER, D., MAYER, R. & WIEAND, H. (1993). An intergroup trial of intensive course 5-FU and low dose leucovorin as surgical adjuvant therapy for high risk colon cancer. *Proc. Am. Soc. Clin. Oncol.*, **12**, 190.

SAMAL, B.A., FOULKES, M.A. & MCDONALD, B. (1984). Levamisole probably shortens response and survival in CMFP maintained advanced breast cancer patients. *Proc. Am. Soc. Clin. Oncol.*, **3**, 126.

SERTOLI, M.R., GUARNERI, D. & RUBAGOTTI, A. (1987). Adjuvant immunochemotherapy in colorectal cancer Dukes C. *Oncology*, **44**, 78–81.

STEVENSON, H.C., GREEN, I., HAMILTON, J.M., CALABRO, B.A. & PARKINSON, D.R. (1991). Levamisole: known effects on the immune system, clinical results, and future applications to the treatment of cancer. *J. Clin. Oncol.*, **9**, 2052–2066.

TREURNIET-DONKER, A.D., MEISCKE DE JONGH, M.L. & VAN PUTTENS, W.L.J. (1987). Levamisole as adjuvant immunotherapy in breast cancer. *Cancer*, **59**, 1590–1593.

VERHAEGEN, H. (1978). Postoperative levamisole in colorectal cancer. In *Immunotherapy of Malignant Disease*, Rainer, T. (ed.) pp. 94–100.

VERHAEGEN, H., DECREE, J. & DECOCK, W. (1982). Levamisole therapy in patients with colorectal cancer. In *Immunotherapy of Human Cancer*, Terry, W.D. & Rosenberg, S.A. (eds) pp. 225–229.

WINDLE, R., BELL, P.R.F. & SHAW, D. (1987). Five year results of a randomized trial of adjuvant 5-fluorouracil and levamisole in colorectal cancer. *Br. J. Surg.*, **74**, 569–572.

WOLMARK, N., ROCKETTE, H., FISHER, B., WICKERHAM, D.L., REDMOND, C., FISHER, E.R., JONES, J., ELEFTHERIOS, P.M., ORE, L., PETRELLI, J., SPURR, C.L., DIMITROV, N., ROMOND, E.H., SUTHERLAND, C.M., KARDINAL, C.G., DEFUSCO, P.A. & JOCHIMSEN, P. (1993). The benefit of Leucovorin-modulated 5-FU as postoperational adjuvant therapy for primary colon cancer: results from National Surgical Adjuvant Breast and Bowel Project Protocol C0-3. *J. Clin. Oncol.*, **11**, 1879–1887.

ZANIBONI, A., ERLICHMAN, C., SEITZ, J.F., SEPHERD, L., MILAN, C., LABIANCA, R., TORRI, V., PIGNON, J.P., ZEE, B. & MARSONI, S. (1993). FUFA increases disease-free survival (DFS) in resected B2 and C colon cancer (CC): results of a prospective pooled analysis of 3 randomized trials (RCTs). *Proc. Am. Soc. Clin. Oncol.*, **12**, 191.